# Amino acid modified [70] fullerene derivatives with high radical scavenging activity as promising bodyguards for chemotherapy protection

**DOI:** 10.1038/s41598-018-34967-7

**Published:** 2018-11-08

**Authors:** Yue Zhou, Mingming Zhen, Mirong Guan, Tong Yu, Liang Ma, Wei Li, Jiasheng Zheng, Chunying Shu, Chunru Wang

**Affiliations:** 1 CAS Key Laboratory of Molecular Nanostructure and Nanotechnology, and Beijing National Laboratory for Molecular Sciences, CAS Research/Education Center for Excellence in Molecular Sciences, Institute of Chemistry, Chinese Academy of Sciences, 100190 Beijing, China; 2 University of Chinese Academy of Sciences, 100049 Beijing, China; 3 Center of Interventional Oncology and Liver Diseases, Beijing You’an Hospital, Capital Medical University, 100069 Beijing, China

## Abstract

Despite the great efforts for tumor therapy in the last decades, currently chemotherapy induced toxicity remains a formidable problem for cancer patients, and it usually prohibits the cancer therapy from successful completion due to severe side effects. In general, the main side effects of chemotherapeutic agents are from the as-produced reactive oxygen species (ROS) that not only harm the tumor cells but also damage the patients’ organs. Here we report the application of amino acid derivatives of fullerene (AADF) in the chemotherapy which strongly scavenge the excess ROS to protect the tested mice against the chemotherapy-induced hepatotoxicity and cardiotoxicity. Two amino acids, i.e., L-lysine and β-alanine were separately employed to chemically modify C_70_ fullerene, and L-lysine derivative of fullerene (C_70_-Lys) exhibits superior radical scavenging activity to β-alanine derivative of C_70_ (C_70_-Ala). As expected, C_70_-Lys show much better protective effect than C_70_-Ala against the chemotherapy injuries *in vivo*, which is verified by various histopathological, haematological examinations and antioxidative enzyme studies. Moreover, the L-glutathione level is increased and the cytochrome P-450 2E1 expression is inhibited. They are potentially developed as promising bodyguards for chemotherapy protection.

## Introduction

Chemotherapy is currently the mainstream for clinical cancer treatments, but the severe side effects of chemotherapeutic drugs are extremely hard to reduce^1–3^. Doxorubicin abbreviated as DOX, an anthracycline antibiotic, is one of most effective chemotherapeutic agents for a wide variety of cancer treatments^4–6^. However, the clinical application of DOX is limited due to its acute and chronic adverse effects on liver, heart, kidneys and so on^7^. It has been reported that the generation of excessive free radicals during DOX chemotherapy contributes to these side effects^8,9^, especially the serious hepatotoxicity^10^ and cardiotoxicity^11^. Certain free radicals know as reactive oxygen species (ROS) are considered to be an important factor for oxidative stress and damage, which leads to the imbalance of oxidant-antioxidant systems^12,13^. And a clinically strategy is to employ antioxidant agents simultaneously to alleviate the injury induced by oxidative stress^14,15^. Thus, it is of great significance to seek a chemical species with a high efficiency to scavenge free radicals, both for preserving our health and for utilization of cancer chemotherapy.

Fullerene, since its discovery in 1985, has been extensively studied in the biomedical field including its great potential in free radical scavenging^16,17^. The plenty of conjugated double bonds and the low lying lowest unoccupied molecular orbital (LUMO) of fullerenes give them the ability to efficiently capture electrons. Due to the insolubility of unmodified fullerenes in water, diverse chemical modifications have been applied to improve their water solubility and biocompatibility, such as the carboxyl-modified^18^, hydroxylated (known as fullerenols)^19^, ethylenediamine-modified^20^ and amino acid modified fullerenes and metallofullerenes^21^, all of which exhibit different degrees of antioxidant activities *in vitro* or *in vivo*. For example, we have recently reported another water-soluble gadofullerene nanoparticles, Gd@C_82_-(EDA)_8_ based on ethylenediamine (EDA)-modified Gd@C_82_, which revealed a better cytoprotective effect on human epidermal keratinocytes-adult cells than Gd@C_82_(OH)_26_ nanoparticles^20^. Chen *et al*.^22^ synthesized three kinds of water-soluble fullerenes (C_60_(C(COOH)_2_)_2_, C_60_(OH)_22_ and Gd@C_82_(OH)_22_) and demonstrated that both the surface chemistry and physical properties would influence their biological and biomedical activities. In addition, some other hydrophilic fullerene derivatives have also been studied for their protection against chemotherapy medicines induced free radical damage^23,24^.

In this work, two water-soluble amino acid derivatives of fullerene have been prepared by a simple one-step reaction through the nucleophilicity of nitrogen atoms towards the carbon cage. In consideration of the possible mechanism of the reaction between amino acids and fullerenes, we speculate that the amino group plays a key role in the reaction. L-lysine and β-alanine were chosen for their simple and typical structures, which would not cause the steric hindrance with fullerene cage during the reaction. L-lysine is susceptible to react with fullerene compared to β-alanine owing to one extra amino group. We investigated the chemical and physical properties of the derivatives and discovered that the L-lysine derivative of fullerene (C_70_-Lys) exhibited better capability in scavenging ROS than the β-alanine derivative fullerene (C_70_-Ala). Furthermore, for the hepatoprotective and cardioprotective effects against DOX, as well as the cytoprotective effect on human umbilical vein endothelial cells (HUVECs), the C_70_-Lys were also more excellent than that of the C_70_-Ala, which attributed to the different modifications on the fullerene surface.

## Material and Methods

### Chemicals

Solid C_70_ (99% purity) was obtained from Xiamen Funano Co. Ltd., China. L-lysine, β-alanine, 5,5-dimethyl-1-pyrroline-N-oxide (DMPO), doxorubicin and vitamin C (VC) were purchased from Sigma-Aldrich (St. Louis, MO, USA). Hydrogen peroxide (H_2_O_2_) was obtained from Chemical Reagents Co., Beijing. The assay kits of ALT, AST, LDH, CK andα-HBDH were purchased from Toshiba Medical Systems Corporation. The assay kits of MDA, GSH, GSSG, GSH-Px, GR, CAT and SOD to detect the oxidative status in organs were obtained from Nanjing Jiancheng Bioengineering Institute (Nanjing, China). All reagents and solvents were obtained commercially and used without further purification.

### Preparation of water-soluble amino acid derivatives of fullerene (AADF)

Highly purified L-lysine and β-alanine substitute derivatives of C_70_ were prepared by a method similar to Shu’s^21^. Briefly, a solution was prepared by dissolving NaOH (0.412 g, 10 mmol) and β-alanine (0.868 g, 10 mmol) or L-lysine (1.462 g, 10 mmol) in deioned water (3 mL), and then ethanol (10 mL) was added. The resulting solution was added dropwise to 30 mL C_70_ (ca. 50 mg) toluene solution under stirring conditions, and the solution was stirred at 80 °C for ca. 24 h (for L-lysine) or 36 h (for β-alanine) until the organic layer became completely colorless. After reaction the aqueous layer was separated from organic layer, concentrated, and dialyzed against deionized Milli-Q water (Millipore, Billerica, MA, USA) for 3 days before freeze drying.

### Characterization of water-soluble AADF

Solid AADF were obtained after freeze drying, then characterized by elemental analysis (EA), thermogravimetric analysis (TGA) (Shimadzu DTG-60H, Japan), dynamic light scattering (DLS) and zeta-potential measurements. TGA was conducted under a nitrogen flow (50 mL/min) and the samples were heated from room temperature to 500 °C at a ramp rate of 5 °C/min. DLS and zeta-potential measurements were performed on a NanoZS ZEN3600, Malvern instrument at 25 °C. All of the samples were measured three times in Milli-Q water at pH 7.0 after filtering through a 0.22 μm pore size membrane.

### Cryo-TEM characterization

Samples for cryo-TEM were prepared with a Leica EM GP immersion freezer (Leica Microsystems) as follows: 3 μL of each sample was loaded onto a glow discharged carbon-coated holey TEM gird (GIG) and blotted with a filter paper to obtain a thin liquid film on the grid. Then the grid was quickly plunged into liquid ethane (cooled by liquid nitrogen). The vitrified samples were then transferred to a cryogenic sample holder (626, Gatan) and examined with a TEM (JEM-2010, JEOL) at 94 K. Micrographs were captured with a CCD camera (Gantega, Olympus Soft Imaging Solutions).

### Cell viability assessment

Cell viability was evaluated by the activity of mitochondrial dehydrogenase according to a protocol previously described^20^. In brief, to detect the pre-protection of AADF against DOX-induced oxidative stress, the HUVECs were cultured with different concentrations (100, 300, 500, 800, 1000 ug/mL) of AADF in 96-well plates for 3 h separately, and then treated with 4 μg/mL DOX for 1 h. Between DOX and AADF, the cells were washed with phosphate buffer saline (PBS) to remove the residue. After that, the medium was replaced with fresh medium and incubated in the dark at 37 °C for 24 h. A WST-8 assay with a Cell Counting Kit-8 (CCK-8; DOJINDO, Kumamoto, Japan) was used to evaluate the cell viability. The absorption value at 450 nm was read using a 96-well plate reader (iMark microplate reader, Bio-RAD, USA). Each treatment was repeated three times. For the post-repair of AADF evaluation, the HUVECs were cultured with DOX for 1 h first and followed by the incubation with AADF for 3 h. Other processing kept consistent with the pre-protection action. Experiments were performed at least three times.

### Scavenging of hydroxyl radicals

Interception of hydroxyl radicals by AADF was determined by electron paramagnetic resonance (EPR) (JEOL, JEF FA200, Japan). Hydroxyl radicals were generated by H_2_O_2_ with the irradiation of ultraviolet light (UV), and DMPO, as a radical capturer, could immediately transfer them to DMPO-OH which would be detected by EPR. A solution containing 100 μL of DMPO (100 mM) and 50 μL of H_2_O_2_ (1 M) was mixed with 50 μL of ultrapure water, C_70_-Lys (100 μg/mL), and C_70_-Ala (100 μg/mL), separately. The solutions were firstly irradiated with a 500 W UV lamp for 4 min, and then recorded the X band EPR spectrum in the dark. The scavenging effect was judged by comparison with the control group lacking AADF.

### Animals and treatments

Six-week-old female BALB/c mice with body weights in range of 18.0 g to 20.0 g were purchased from Center for Experimental Animals, Institute of Process Engineering, Chinese Academy of Sciences (Beijing, China). All animal experiments complied with the protocols approved by the National Regulation of China for Care and Use of Laboratory Animals. Mice were randomly distributed into five groups (n = 6) as follows: 1) control healthy group treated with saline only every day; 2) DOX + saline group; 3) DOX + VC group; 4) DOX + C_70_-Lys group; 5) DOX + C_70_-Ala group. The latter four groups were treated with intraperitoneal (*i.p*.) injection of saline, VC (10 mg/kg/day) and AADF (10 mg/kg/day) once a day for eleven days, respectively. The choice for effective dosage was investigated by detecting a short-term change of haematological parameters and organ coefficients. And at the 4th day all the mice (except control healthy group) received a single intravenous injection (*i.v*.) of DOX 30 minutes after the injection of saline, VC or AADF. DOX was injected at a dose of 20 mg/kg, which would induce cardiotoxicity and hepatotoxicity in mice.

### Coefficients of liver and heart

The coefficients of liver and heart to body weight were measured by weighing the organs and calculating the ratio of organ tissues (wet weight, mg) to body weight (g).

### Hematology examination

By quickly removing the eyeball on the eleventh day, the blood samples were divided into two parts, one for blood routine index examination and the other centrifuged for biochemical analysis. Some blood routine indexes were measured by a hematology analyzer (BC-5000VET, Shenzhen, China), including WBC, RBC, HGB, HCT, PLT, neutrophil and lymphocyte. The second portion was used to evaluate the toxicity towards liver and heart by measuring serum levels of ALT, AST, LDH, CK and α-HBDH, using an automated biochemical analyzer (TBA-2000FR, Tochigi, Japan).

### Biomarker assay of liver and heart tissues

Each liver and heart was quickly removed from the sacrificed mice and placed into liquid nitrogen for the analysis of enzymatic activity. They were minced and homogenized for the measurements of MDA and the assays of total protein concentration, LDH, GSH, GSSG, GSH-Px, GR, CAT and SOD. The levels of LDH, GSH and GSSG were measured by an automatic microplate reader (MULTISKAN MK3, Thermo, USA). And the spectrophotometry was used to determine the activities of GSH-Px, GR, CAT and SOD (UV-2000, Jinghua, China).

### Histopathological examination

Immediately after surgical removal, the liver and heart tissues were harvest and fixed in a 4% paraformaldehyde solution. Hematoxylin and eosin staining was conducted based on the standard manual. In brief, the tissues were immersed in paraffin blocks, sliced into 5 μm sections, unfolded on the histological slides for staining. Histological observations and photomicrography of normal tissue slides were performed using an optical microscope and the sections of liver/heart were scanned by a scanner (Magscanner, KF-PRO-005, Japan).

### Western blotting

Western blotting was performed to determine the protein expression of CYP2E1. A 200 μg portion of each precleared, detergent-solubilized liver and heart tissues was used for total protein preparation. Bicinchoninic Acid (BCA) protein assay (Thermo Fisher Scientific, USA) was used to determine protein concentration. All operations were carried out in accordance with the manual specification.

### Statistical Analysis

All data presented are means or means ± standard deviations. According to a Student’s t-test the variance in different groups was statistically analyzed to evaluate its significance and *P* < 0.05 were recognized as statistically significant (*) and *P* < 0.01 were greatly statistically significant (**).

## Results and Discussion

### Preparation and Characterization of AADF

**Figure 1:**
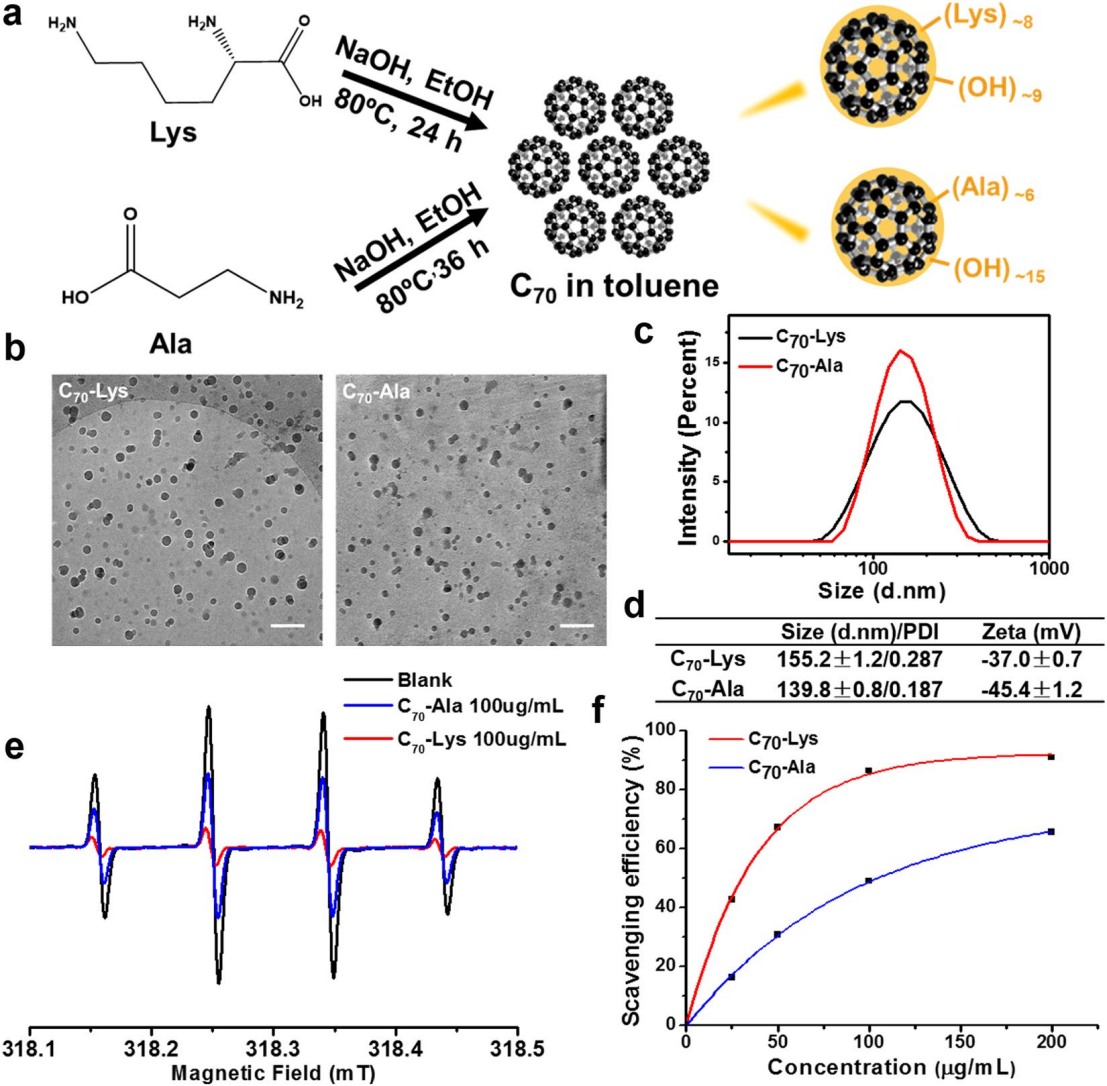
(**a**) Schematic illustration of the preparation of AADF *via* a facile one-step synthetic method. (**b**) Cryo-TEM micrographs of AADF. Scale bar is 200 nm. (**c**) Hydrodynamic size distributions of AADF in aqueous solution. (**d**) Comparison of Z-average sizes and the zeta potentials of AADF. (**e**) X-band EPR spectra of the hydroxyl radicals captured by DMPO after treatment with 100 μg/mL C_70_-Ala and C_70_-Lys. Ultrapure water was used as a blank. (**f**) Scavenging efficiency with the concentration increase of AADF measured by EPR.

Both of C_70_-Lys and C_70_-Ala were simply prepared by a facile one-step synthetic method with the addition of alkali and ethanol under heating condition (Scheme in Fig. 1a). With the higher number of amino group in L-lysine than that of β-alanine, L-lysine is susceptible to react with the carbon cage and needs less reaction time for the synthesis of C_70_-Lys. The obtained aqueous layers after reaction were concentrated and further purified by dialysis. It was well known that the conventional characterization methods for small molecules were not suitable for our fullerene derivatives. Thus a previously established method^25,26^, the elemental analysis along with a study for hydration water by TGA, was conducted to estimate their average structures (Table S1 and Fig. S1). The ratio of nitrogen to carbon (N/C) from the elemental analysis was used to assess the average number of amino acid group, approximately with eight L-lysine and six β-alanine molecules severally attached to the fullerenes. According to the TGA measurements, C_70_-Lys and C_70_-Ala showed 9.9% and 12.4% weight loss before 150 °C, respectively, which was the degradation of physically absorbed and hydration water. The subsequent loss of the samples was ascribed to the detachment of the polyol moieties and amino acid groups from fullerenes. Combined with the hydrogen contents of elemental analysis, we got the average numbers of hydroxyl group, nine for C_70_-Lys and fifteen for C_70_-Ala, respectively. Therefore, the average structures of the two samples were deduced as C_70_(OH)_x_(NH(CH_2_)_4_CHNH_2_COOH)_y_ (x ≈ 9, y ≈ 8) and C_70_(OH)_m_(NHCH_2_CH_2_COOH)_n_ (m ≈ 15, n ≈ 6).

Cryo-TEM characterization of AADF has indicated their sizes in aqueous solution. Both of them tend to aggregate into nanoparticles with a wide distribution (20–70 nm), and the diameter of C_70_-Lys is larger than C_70_-Ala (Fig. 1b). The DLS investigation shows that the hydrodynamic diameters of AADF in aqueous solution are 155.2 nm (C_70_-Lys) and 139.8 nm (C_70_-Ala) (Fig. 1c), which accords with the result of Cryo-TEM. Both of them revealed a single peak, which was a stable form driven by the aggregation behavior and would be possible for cellular uptake. They revealed high stability in physiological mediums without forming any aggregation even after centrifugation at 8000 rpm for 10 min (Fig. S2). It is worth mentioning that the zeta potential of C_70_-Lys (−37.0 mV) was more positive than that of C_70_-Ala (−45.4 mV), implying successful introduction of L-lysine onto C_70_ and exposing more amino groups outside (Fig. 1d). The distinctions in structure and zeta potential between these two samples would greatly influence their physicochemical properties, especially the radical scavenging ability and the behaviors at cellular level as discussed below.

### Hydroxyl radical scavenging capability

Hydroxyl radicals (^•^OH), the most potent free radical produced from oxygen, are extremely powerful and active oxidants in the presence of biologic substrates, damaging their nuclear and mitochondria DNA, membrane lipids and carbohydrates^27,28^. We chose hydroxyl radical as a representative to evaluate the radical scavenging capability of AADF by the EPR method. The effects of the two kinds of AADF on the EPR spectra of ^•^OH are shown in (Fig. 1e,f, Fig. S3). The intensities of the DMPO-OH adduct were reduced by addition of C_70_-Lys or C_70_-Ala, indicating both of them possessing the radical scavenging capability. The radical scavenging properties of lysine and alanine were detected by the EPR method, which didn’t show any capability in scavenging free radicals (Fig. S4). At the same concentration (100 μg/mL), C_70_-Lys scavenged about 87% of the generated hydroxyl radicals compared with reduction in signal intensity of 49% for C_70_-Ala. We attributed the different efficiency of scavenging abilities to the distinction of the fullerene cage integrity in accord with the structural properties of AADF. A better conjugation provided more reactive sites to attack the ROS, thus enhanced the efficiency of scavenging reactive species.

### Protective effects of AADF against DOX-induced oxidative stress using HUVECs

**Figure 2:**
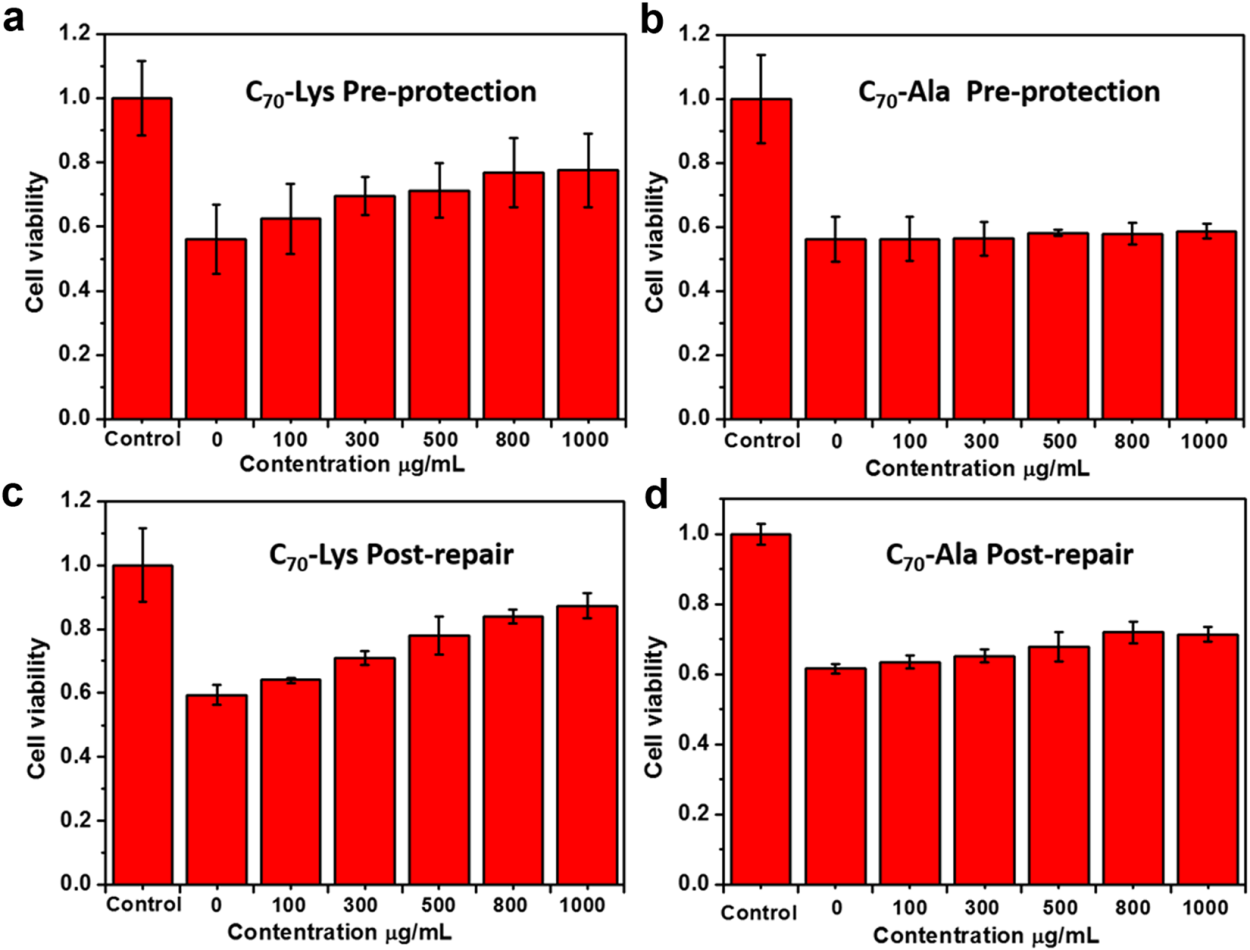
(**a**,**b**) The pre-protective effects of C_70_-Lys (**a**) and C_70_-Ala (**b**) against DOX-induced damage in HUVECs. The cells were cultured with 100–1000 μg/mL AADF separately for 3 h before incubation with DOX for 1 h. Cells treated with only PBS were used as a control. (**c**,**d**) The post-repair effects of C_70_-Lys (**c**) and C_70_-Ala (**d**) against DOX-induced damage in HUVECs. The operation kept alliance with the above except changing the incubation order of AADF and DOX.

Before the evaluation of the protective effects, we have investigated the cytotoxicity of the samples toward HUVECs by incubation with AADF for 24 h (Fig. S5). Their negligible toxicity is an absolute and obvious prerequisite for their potential use in biomedicine. The protective effects of the two AADF against DOX-induced oxidization were investigated using HUVECs. For the study of pre-protection, the cells were treated with AADF for 3 h before the incubation with DOX for 1 h. While for the post-repair test, we exchanged the order of AADF and DOX treatments. As shown in Fig. 2, the concentration dependent protection and repair were observed and C_70_-Lys exhibited an apparent protective effect compared with C_70_-Ala. As the concentration increasing from 100 to 1000 μg/mL, the cell viability treated with C_70_-Lys displayed a steady rise, with respect to the mild increase of that treated with C_70_-Ala (pre-protection ratio: C_70_-Lys 21.5% versus C_70_-Ala 2.5%; post-repair ratio: C_70_-Lys 27.9% versus C_70_-Ala 10.4%). AADF also showed their superioty to C_70_ suspensions, which may be ascribed to the poor dispersity of C_70_ suspensions (Fig. S6). This result demonstrated a correlation between the cellular protective effects and their radical scavenging capability.

### Protective effects of AADF against DOX-induced hepatotoxicity and cardiotoxicity *in vivo*

**Figure 3:**
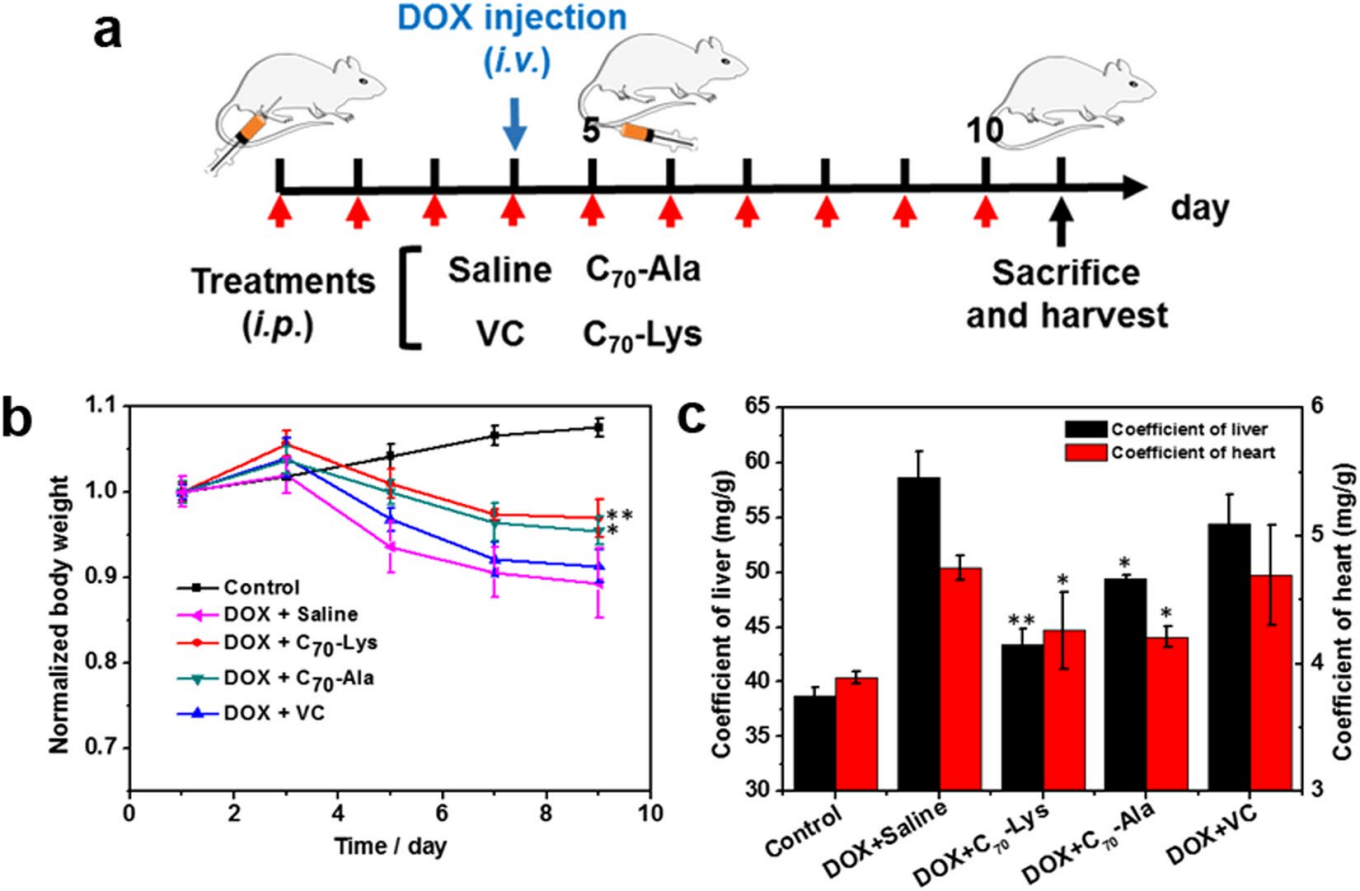
(**a**) Protective treatment schedule of AADF in DOX-induced hepatotoxicity and cardiotoxicity in mice. (**b**) Body weight curves after various treatments indicated. (**c**) The coefficients of liver and heart after sacrificing. ^*^P < 0.05 and ^**^P < 0.01 *vs*. DOX + saline group.

The DOX-induced toxicities towards liver and heart have been widely reported^29,30^, thus we choose DOX as a chemotherapeutic agent to investigate *in vivo* the potential hepatoprotective and cardioprotective effects of AADF during the chemotherapy. Firstly, we set two groups of mice treated with C_70_-Lys or C_70_-Ala for 7 days. We tested the haematological parameters (neutrophil and lymphocyte, the indicator of inflammation) at 3th and 7th day (Table S2), and the histopathological examinations of the main organs (heart, liver, spleen, lung and kidneys) were conducted by H&E staining (Fig. S7). Neither C_70_-Lys nor C_70_-Ala showed any toxicity towards the mice. For system study, thirty BALB/c mice were randomly distributed into five groups (n = 6): control healthy group, DOX + saline group, DOX + VC group, DOX + C_70_-Lys group and DOX + C_70_-Ala group. The control healthy group was treated with saline only and all the other four groups were received intravenously (*i.v*.) application of DOX (20 mg/kg of body weight) at the fourth day after the pre-treatments of saline, VC, C_70_-Lys or C_70_-Ala for 3 days respectively. Then they continued to be intraperitoneally (*i.p*.) injected with the drugs in the following six days (scheme Fig. 3a). The optimal dosage of AADF was investigated ahead by detecting a short-term change of haematological parameters (Table. S3) and organ coefficients (Table. S4). We chose the concentration of 1.0 mg/mL for system studies *in vivo*. Allowing for high toxicity usually results in weight loss, the body weight of each mouse was checked every other day during the treatments. For the first three days before the injection of DOX, the body weights of all the mice exhibited a rapid growth, and among them the group treated with C_70_-Lys had the fastest growth rate. Besides, the DOX + C_70_-Lys and DOX + C_70_-Ala groups showed a greater resistance to the toxicity caused by the DOX based on the decay rate of weight (Fig. 3b). By comparison, the group treated with VC, slightly resisted the toxicity induced the drop of body weight. The final body weights of the DOX-treated mice were more than 10% less with respect to the control healthy group. And significant different values of the coefficients for liver and heart were found between the AADF-treated groups and saline-treated group (Fig. 3c). For the AADF-treated groups, the coefficients were obviously lower compared with the DOX + saline group, implying the AADF had a certain improving effect on the hepatomegaly and enlarged hearts caused by DOX. This macroscopic variation has a good correlation with the symptom in DOX-induced liver and heart injury previously reported^10,31^.

**Figure 4:**
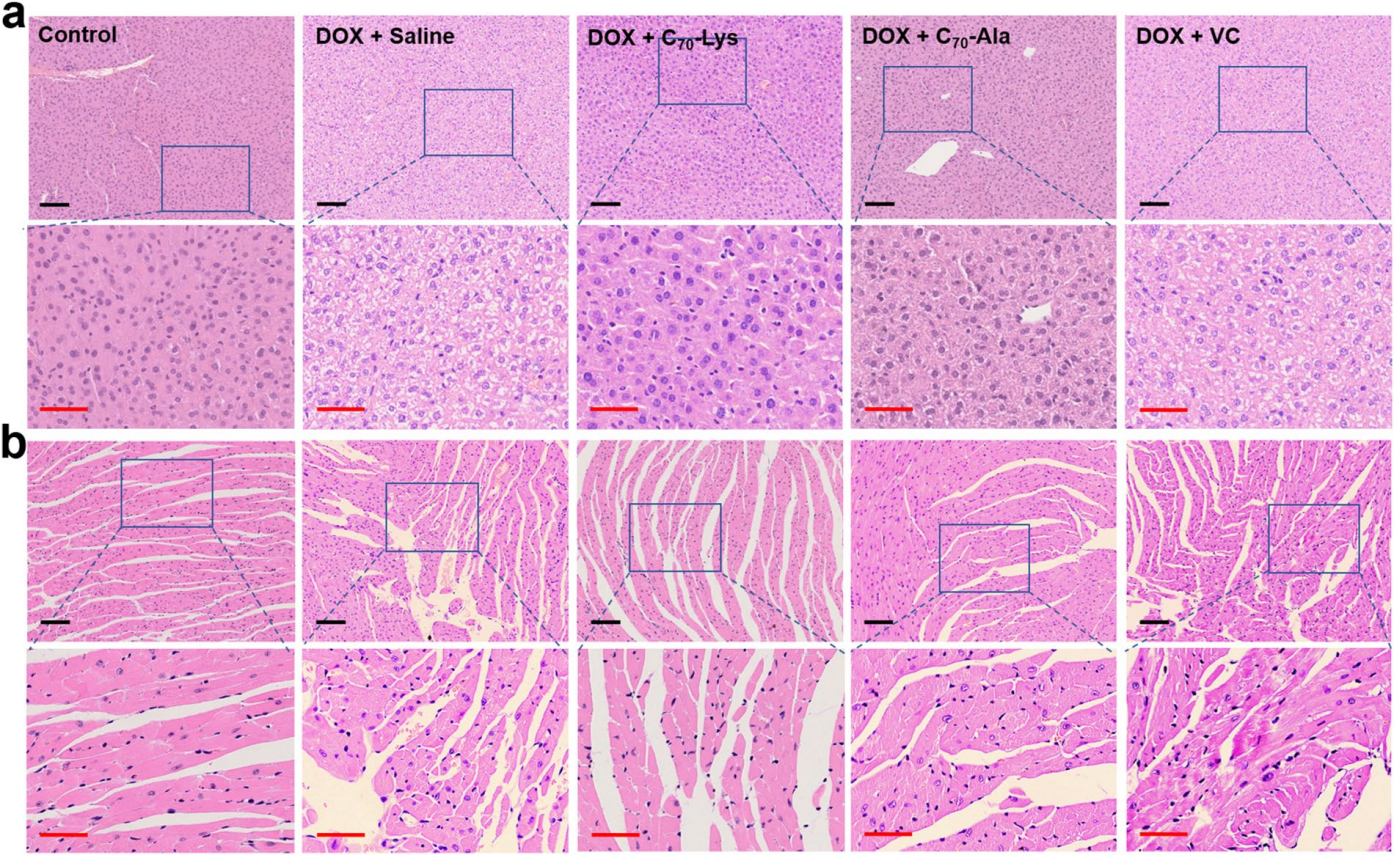
The histologic sections of the liver (**a**) and heart (**b**) of the mice in the control group, DOX + saline group, DOX + C_70_-Lys group, DOX + C_70_-Ala group, DOX + VC group. Scale bars are 100 μm (in black) and 50 μm (in red).

The histopathological examinations of the liver and heart were studied using hematoxylin&eosin (H&E) stained tissue slices. Compared with the control group, the DOX induced extensive and severe lesions, classified as hepatocellular ballooning degeneration, vacuolization of cytoplasm and single cell necrosis in the liver tissues (Fig. 4a). It also caused the interstitial myocardial edema, disordered arrangement of myocardial fibers with vesicular degeneration and loose cytoplasm of myocardial cell in the heart tissues (Fig. 4b). These histopathological changes qualitatively agreed with the macroscopic variation of the liver and heart coefficients, which implied the swelling of the organs treated with DOX. Although slight vacuolization of cytoplasm was observed in DOX + C_70_-Lys group and DOX + C_70_-Ala group, the degeneration of hepatocytes and myocytes induced by DOX were significantly suppressed. The protection of AADF from histologic observation reflected its advantage over the traditional antioxidant, VC. It’s worth mentioning that there were almost no hepatocellular ballooning degeneration and disordered arrangement of myocardial fibers in the mice treated with AADF.

We collected the blood samples to measure haematological parameters and some markers of cellular injury in serum. From the results of white blood cells (WBC), red blood cells (RBC), hemoglobin (HGB), hematocrit (HCT), blood platelet (PLT), neutrophil and lymphocyte, a positive influence of AADF was found on the abnormality of haematological parameters after treatment with DOX (Table 1). And significant variation in almost all parameters was noted for the C_70_-Lys and saline treated DOX groups, especially the neutrophil and lymphocyte which represented the inflammation. For the healthy mice, about 80% of WBC were lymphocytes and 17% were neutrophils. It was obvious that the DOX treatment caused a decrease of lymphocyte and an increase of neutrophils. The neutrophil, a first responder to the xenobiotic, usually raised number as the acute inflammatory processes^31^. The level of producing neutrophils of AADF-treated groups was lower than that of the DOX + saline group, which indicated the AADF had an improvement for the injury induced by DOX. The variation of RBC and WBC in groups treated with AADF also showed the positive effects on chemotherapy-induced myelosuppression. Compared comprehensively, DOX + C_70_-Lys group had a better chemoprotective effect than the DOX + C_70_-Ala group.

**Table 1:** Effects of AADF on haematological parameters with DOX-induced hepatoxicity.^*^P < 0.05 and ^**^P < 0.01 *vs*. DOX + saline group.

Parameter	Groups				
	Control	DOX + Saline	DOX + C_70_-Lys	DOX + C_70_-Ala	DOX + VC
WBC (×10^9^/L)	5.31 ± 1.27	4.33 ± 0.72	4.45 ± 0.66^*^	4.36 ± 0.57	4.40 ± 0.99
RBC (×10^12^/L)	7.31 ± 0.95	5.73 ± 0.39	7.15 ± 1.68^**^	6.05 ± 0.92^*^	5.82 ± 0.63
HGB (g/L)	121.25 ± 14.36	95.75 ± 5.74	119.50 ± 9.19^**^	109.67 ± 18.77^*^	97.67 ± 12.02
HCT (%)	39.63 ± 4.30	28.43 ± 2.34	37.55 ± 5.43^**^	33.43 ± 7.09^*^	28.05 ± 2.90
PLT(×10^9^/L)	567.75 ± 87.17	741.67 ± 45.54	582.67 ± 57.74^**^	595.75 ± 52.82^**^	735.25 ± 78.79
Neutrophil (%)	17.60 ± 3.35	22.60 ± 3.52	15.70 ± 3.30^**^	18.75 ± 1.44^*^	24.27 ± 3.93
Lymphocyte (%)	81.82 ± 6.90	73.92 ± 3.67	81.63 ± 4.25^**^	75.86 ± 4.01	72.75 ± 3.17

**Figure 5:**
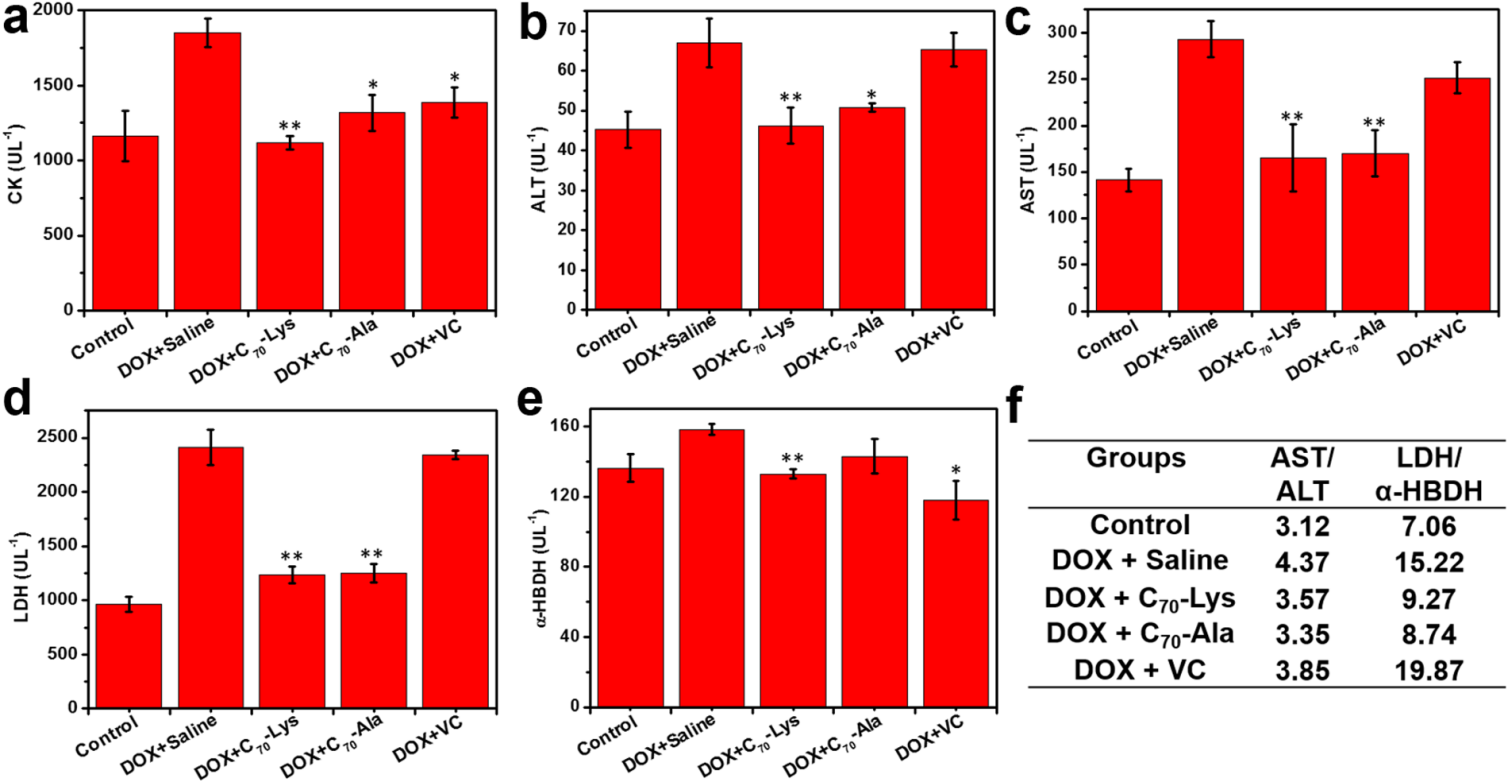
(**a**–**e**) Serum ALT (**a**), AST (**b**), CK (**c**), LDH (**d**), α-HBDH (**e**) levels in the control and DOX treated mice. (**f**) The AST/ALT and LDH/α-HBDH ratios of the groups. ^*^P < 0.05 and ^**^P < 0.01 *vs*. DOX + saline group.

It is well known that creatine kinase (CK), alanine aminotransferase (ALT), aspartate aminotransferase (AST), lactate dehydrogenase (LDH) and α-hydroxybutyrate dehydrogenase (α-HBDH) are usually used as serum enzymatic markers to detect hepatic and cardiac injury^31,32^. ALT and AST are liver-specific enzymes and the ratio of AST to ALT is always used to evaluate the liver status, a high value of the ratio typically indicating the hepatocellular damage^33,34^. According to our results, DOX treatment induced a highly significant increase in serum ALT and AST levels in comparison with the control (Fig. 5a,b). Combined with the ratio of AST/ALT, it could be concluded that the DOX injection had induced marked hepatotoxicity. AST is also a heart-sensitive enzyme and its activity is approximately proportional to the extent of heart injury. In addition, the elevated level of CK, LDH or α-HBDH is commonly the index of myocardial infarction, cardiovascular damage, anaemia or hepatitis^35,36^. With the high level of AST, the increase of CK and LDH activity in the serum of mice treated with DOX is expected (Fig. 5c,d), in accordance with the DOX-induced cardiac damage as previously reported^37^. Administration of AADF markedly lowered the serum ALT and AST levels, as well as the CK and LDH levels, which revealed that the AADF not only had the hepatoprotective but also cardioprotective effects. The α-HBDH levels of the five groups were not statistically different when the mice were sacrificed (Fig. 5e). This phenomenon was also reported by Bjelogrlic^38^ and Injac^36^, which verified the occurrence of a high α-HBDH level within 48 h after an administration of chemotherapeutic agents. With recovery processing in our experimental procedure, the α-HBDH activity had probably dropped to the relatively lower level after one week of DOX treatment. Comparing the AST/ALT and LDH/α-HBDH ratios in Fig. 5f, we demonstrated the advantage of C_70_-Lys over C_70_-Ala in the protection of liver and heart, even better than the commercial liposomal formulations and earlier published fullerene derivative^22^ (Figs S8 and 9).

To further investigate oxidative status in the liver and heart, we tested various parameters of oxidative stress in the liver and heart tissues. Application of DOX significantly raised the activity of all the examined antioxidative enzymes in the liver with regard to the control group (Table 2). Pre- and post- AADF succeeded in preventing the oxidative stress by meaningfully lowered the glutathione oxidized (GSSG), glutathione peroxidase (GSH-Px), glutathione reductase (GR), catalase (CAT) and superoxide dismutase (SOD) levels in different degrees. The improvement of AADF in DOX-caused peroxidative alterations was also evident by the depletion of GSH and elevation of malondialdehyde (MDA) production. GSH exists in all kinds of organs, which can adjust the synthesis of protein and ribonucleotide and is closely related to the antioxidant capacity of the body. MDA is one of the well-known secondary products of lipid peroxidation, and it is widely used as an indicator of cell membrane injury^39^. Their changes had the same trend with the antioxidative enzymes, which once again confirmed that AADF were potential hepatoprotectors against DOX-induced hepatotoxicity. Moreover, fullerene nanoparticles have been reported to own antineoplastic activities by some possible mechanisms, such as angiogenesis inhibition^40,41^, cancer stem cell-specific inhibition^42^ or the regulation of the tumor microenvironment^43^. AADF and DOX may have a synergistic effect to enhance the anti-tumor efficacy, without the impact on the anticancer properties of DOX (Fig. S10).

**Table 2:** Influence of AADF on the indexes of oxidative stress in the liver tissue of the mice.^*^P < 0.05 and ^**^P < 0.01 *vs*. DOX + saline group.

Groups	Control	DOX + Saline	DOX + C_70_-Lys	DOX + C_70_-Ala	DOX + VC
GSH (μmol/g)	22.03 ± 1.44	14.62 ± 3.94	19.61 ± 3.33^**^	17.77 ± 2.93^*^	16.60 ± 3.23
GSSG (μmol/L)	79.95 ± 11.96	111.92 ± 24.18	86.84 ± 12.62^**^	82.61 ± 16.04^**^	97.87 ± 9.35^*^
MDA (nmol/mg protein)	0.94 ± 0.13	2.30 ± 0.29	1.37 ± 0.31^**^	1.37 ± 0.23^**^	1.45 ± 0.24^**^
GSH-Px (U/mg protein)	353.66 ± 53.36	594.30 ± 56.84	334.56 ± 50.26^**^	384.13 ± 32.27^**^	428.31 ± 39.04^*^
GR (U/g protein)	13.40 ± 3.70	22.81 ± 6.61	10.65 ± 2.28^**^	13.99 ± 2.45^**^	16.11 ± 3.62^*^
CAT (U/mg protein)	8.34 ± 1.42	11.53 ± 1.71	6.71 ± 0.41^**^	8.67 ± 1.69^**^	9.15 ± 0.85
SOD (U/mg protein)	95.27 ± 13.98	153.89 ± 13.90	85.34 ± 5.44^**^	97.25 ± 13.79^**^	113.37 ± 11.18^*^

**Figure 6:**
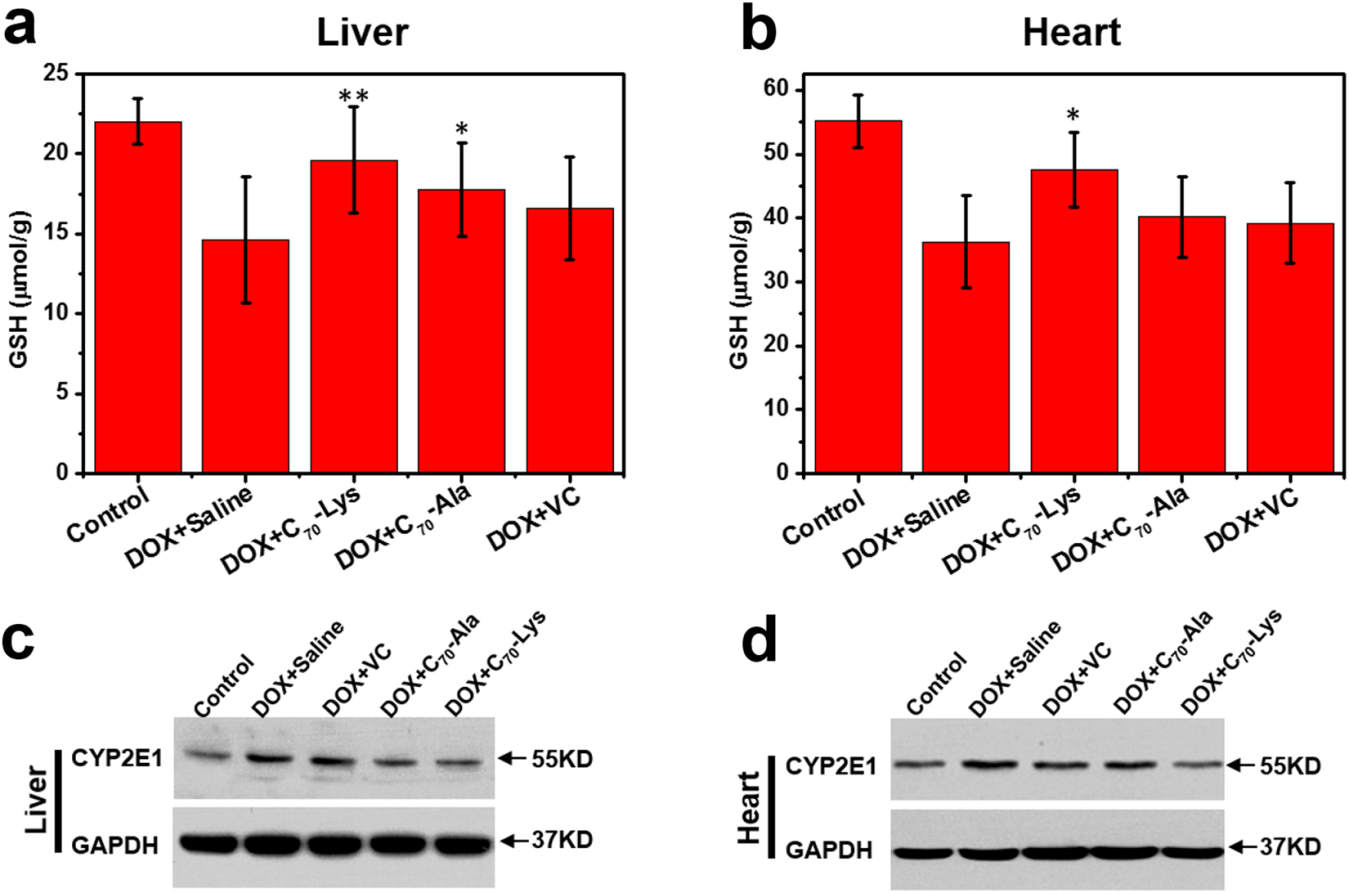
(**a**,**b**) GSH levels in liver and heart of the five groups. (**c**,**d**) CYP2E1 protein expression in the liver and heart. The blots were stripped and probed with the indicated antibody. GAPDH served as loading controls. ^*^P < 0.05 and ^**^P < 0.01 *vs*. DOX + saline group.

To better understand the mechanism relevant with the protection of AADF, the CYP2E1 protein expression was measured by western blotting (WB) analysis. CYP2E1, a membrane protein expressed in high levels in the liver, plays an important role in several metabolic reactions, including the deactivation of endogenous or exogenous harmful substances accompanied by the depletion of GSH^23,44^. When the toxic metabolites exceed the eliminating capacity of GSH, they react with the sulphhydryl groups of protein, resulting in the formation of protein adducts, subsequent mitochondrial dysfunction, oxidant stress, peroxynitrite formation and nuclear DNA fragmentation^45^. As shown in Fig. 6a,b, both the hepatic and cardiac GSH levels of DOX + saline group were greatly higher than that of the control, while the groups treated with AADF were improved in different degree. Associated with the CYP2E1 expression (Fig. 6c,d, Fig. S11), it was ascribed to the modulation of AADF on CYP2E1 expression, thus changing the normal metabolic way of DOX. Notably, AADF were markedly inhibited the hepatic CYP2E1 expression, which can be explained in terms of the high levels of AADF accumulation and CYP2E1 expression in liver.

## Conclusions

We have investigated the impact of different surface modifications on the physicochemical property of fullerene derivatives, as well as the radical scavenging capability. *In vitro* study of cytoprotection against the DOX-induced oxidative stress by using HUVECs demonstrated the superiority of C_70_-Lys compared with C_70_-Ala. We further explored the potential protective effects of AADF in mice with DOX-induced hepatotoxicity and cardiotoxicity, and the distinction between C_70_-Lys and C_70_-Ala in radical scavenging was more distinguishable in the *in vivo* models. These results clearly supported that the chemical functionalization of fullerene plays a significant role in their properties and specific surface modification is great benefit for the biomedical application of these materials.

## Electronic supplementary material

Supplementary Information
