# Supplementary material for: Amino acid modified [70] fullerene derivatives with high radical scavenging activity as promising bodyguards for chemotherapy protection
Source: Sci Rep. 2018 Nov 8;8:16573. doi: 10.1038/s41598-018-34967-7 (PMC6224443; doi:10.1038/s41598-018-34967-7)
Supplement: Supplementary file 1 — Supplementary Information [file 41598_2018_34967_MOESM1_ESM.doc]

**Supplementary Information**

Amino acid modified [70] fullerene derivatives with high radical scavenging activity as promising bodyguards for chemotherapy protection

*Yue Zhou^1,2^, Mingming Zhen^1,2,*^, Mirong Guan^1^, Tong Yu^1,2^, Liang Ma^3^, Wei Li^3^, Jiasheng Zheng^3^, Chunying Shu^1,2^ and Chunru Wang^1,2,*^*

^1^CAS Key Laboratory of Molecular Nanostructure and Nanotechnology, and Beijing National Laboratory for Molecular Sciences, CAS Research/Education Center for Excellence in Molecular Sciences, Institute of Chemistry, Chinese Academy of Sciences, 100190 Beijing, China

^2^University of Chinese Academy of Sciences, 100049 Beijing, China

^3^Center of Interventional Oncology and Liver Diseases, Beijing You’an Hospital, Capital Medical University, 100069 Beijing, China

^*^Correspondence and requests for materials should be addressed to M.Z. (email: zhenmm@iccas.ac.cn) or C.W. (email: crwang@iccas.ac.cn)


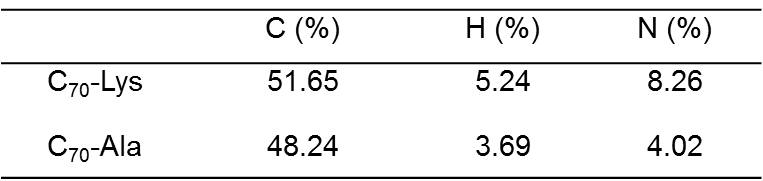


**Table S1.** Elemental analysis of carbon, hydrogen and nitrogen contents in AADF.


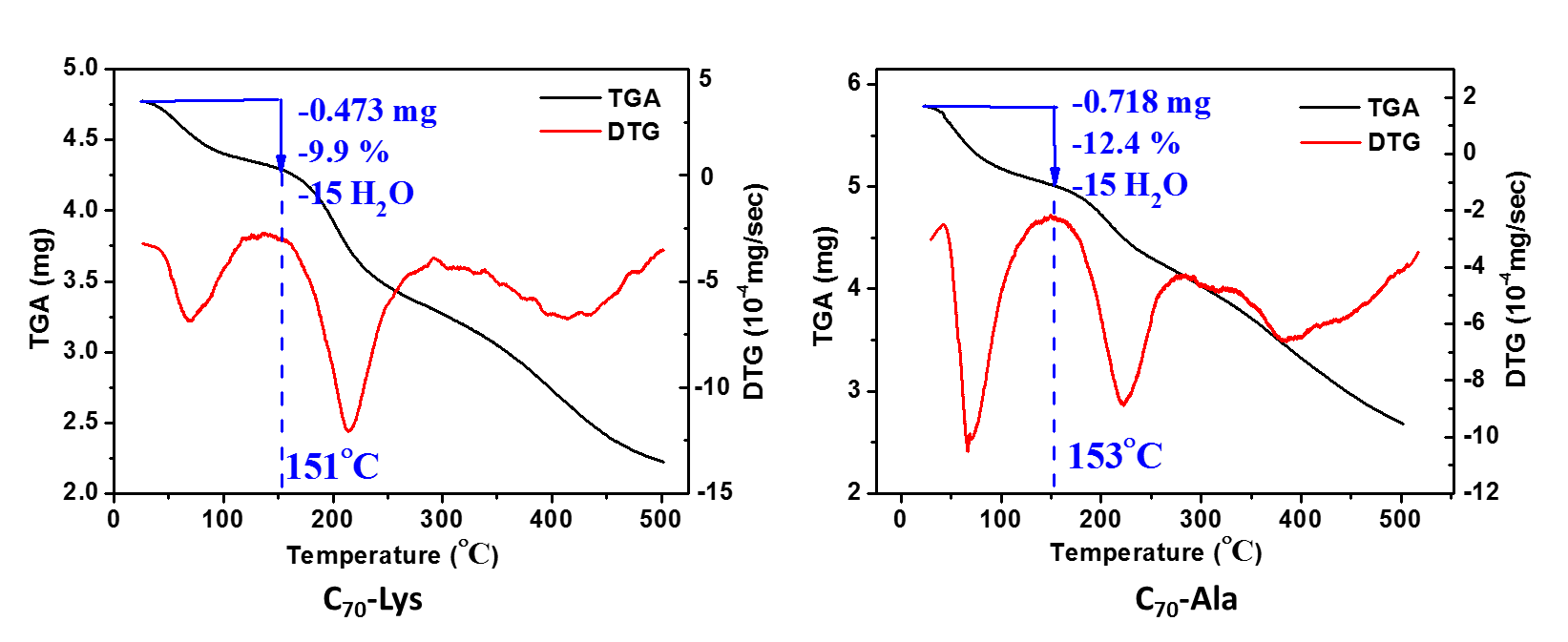


**Figure S1.** Thermogravimetric analysis by a TGA spectrometer to determine the contents of water in AADF.


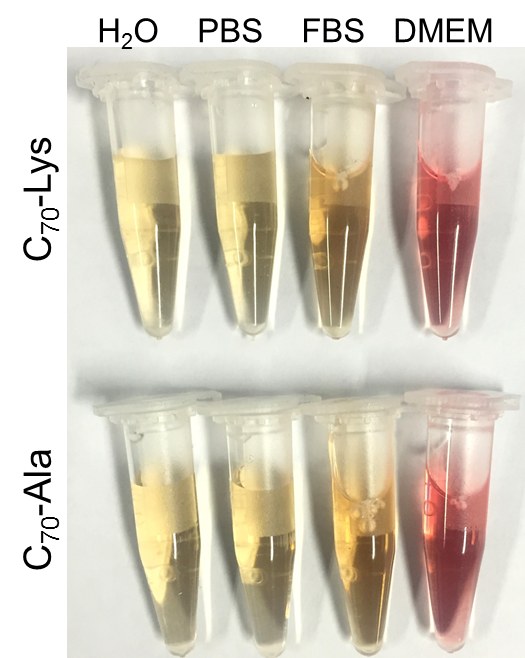


**Figure S2.** Optical imagines of C_70_-Lys and C_70_-Ala in different media after centrifugation at 8000 rpm for 10 min.


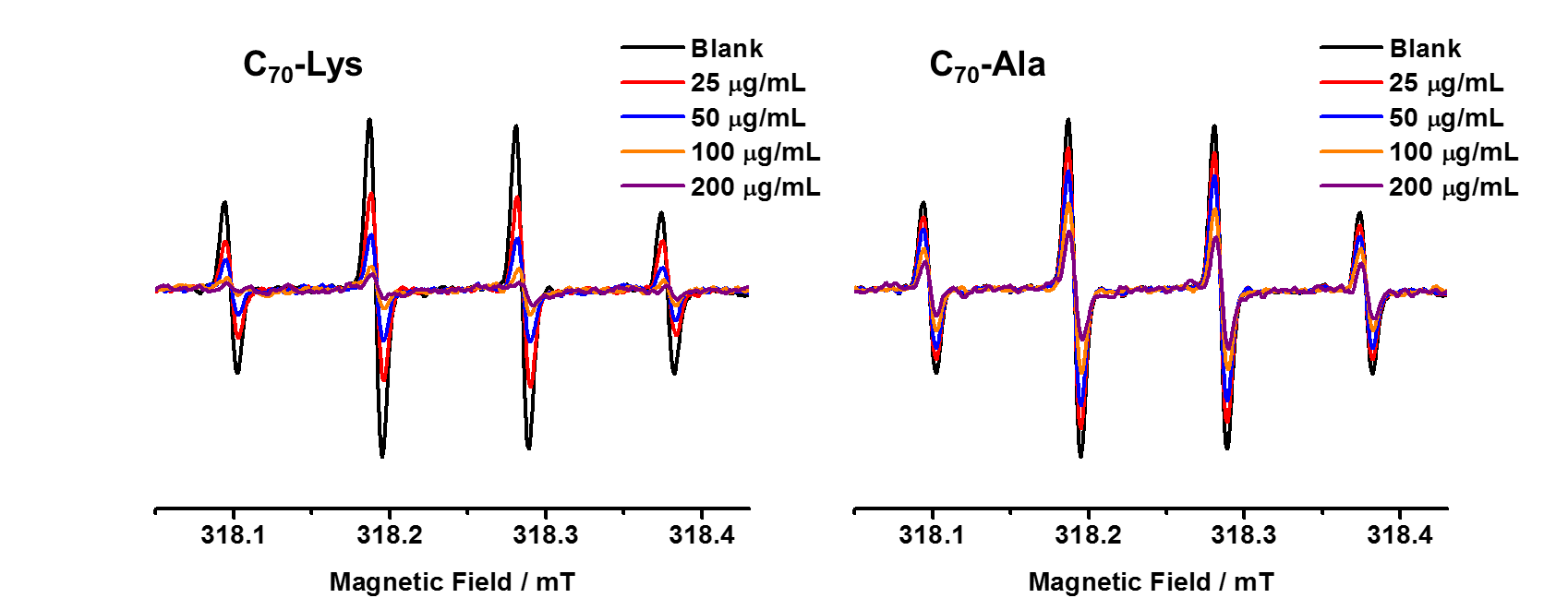


**Figure S3.** X-band EPR spectra of the hydroxyl radicals captured by DMPO after treatment with different concentrations of AADF. Ultrapure water was used as a blank.

**Figure S4.** X-band EPR spectra of the hydroxyl radicals captured by DMPO after treatment with 100 μg/mL lysine and alanine. Ultrapure water was used as a blank. The difference between blank, lysine and alanine is negligible in the margin of error.

**Cell cultures:** Human umbilical vein endothelial cells (HUVECs) were purchased from Shanghai Institute of Cell Biology, Chinese Academy of Sciences (Shanghai, China). Cells were cultured in Dulbecco’s modified Eagle’s medium (DMEM) (Invitrogen, USA) with 10% fetal bovine serum (FBS) (Hyclone Company, South Logan, UT), supplemented with antibiotics (1%, 100 μg/mL, penicillin–streptomycin) (Gibco, Grand Island, N. Y. USA) at 37°C in 5% CO_2_.


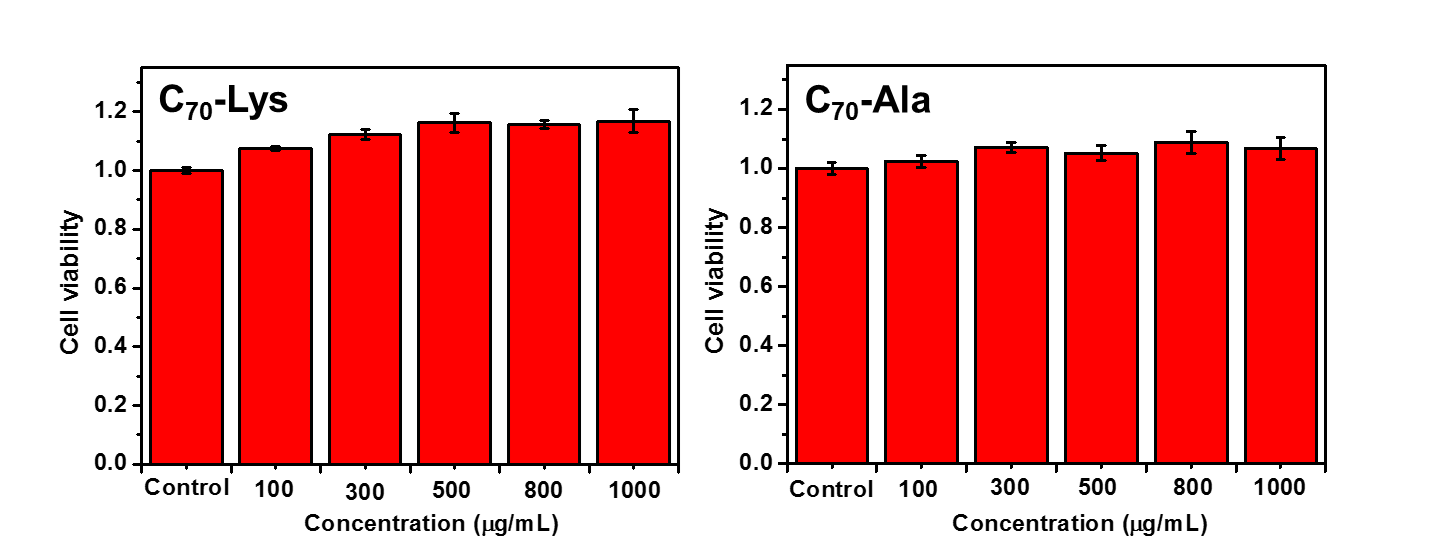


**Figure S5** Cell viability of HUVECs incubated separately with C_70_-Lys or C_70_-Ala at various concentrations (100-1000 μg/mL) in the dark for 24 h. Cells treated with PBS were the control group. After incubation with C_70_-Lys or C_70_-Ala at the relatively high concentration, the cells exhibited higher viability than the control, indicating both of them have negligible cytotoxicity.


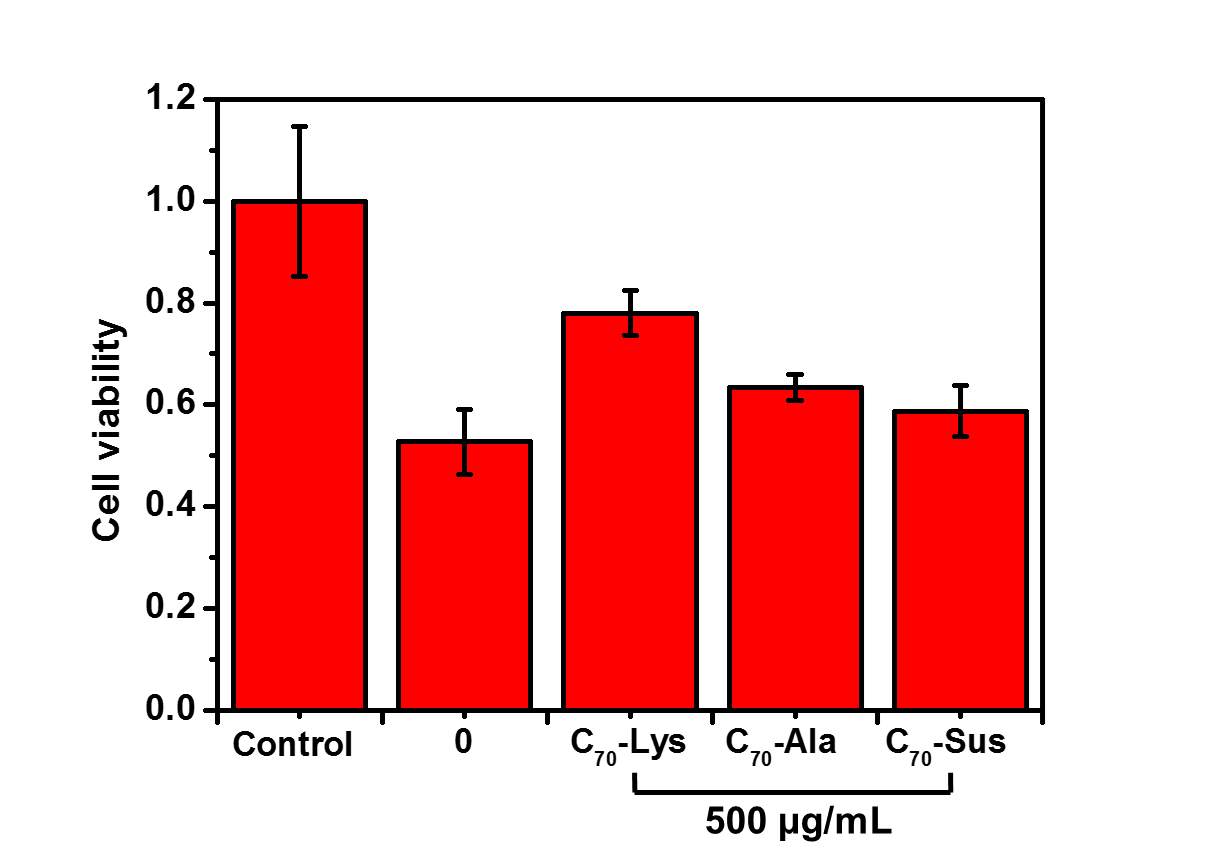


**Figure S6** The post-repair effects of C_70_-Lys, C_70_-Ala and C_70_ suspension against DOX-induced damage in HUVECs.

| Day | Parameter | Group | | |
| --- | --- | --- | --- | --- |
|  |  | Saline | C_70_-Lys | C_70_-Ala |
| Pre | Neutrophil (%) | 18.45 ± 2.05 | 18.30 ± 1.27 | 18.70 ± 1.65 |
|  | Lymphocyte (%) | 79.65 ± 2.35 | 79.80 ± 0.71 | 79.50 ± 0.96 |
| Post-3 d | Neutrophil (%) | 16.37 ± 2.40 | 15.93 ± 0.30 | 18.93 ± 0.65 |
|  | Lymphocyte (%) | 81.17 ± 3.95 | 82.60 ± 1.01 | 79.50 ± 0.46 |
| Post-7 d | Neutrophil (%) | 15.15 ± 0.98 | 14.77 ± 1.18 | 13.93 ± 2.20 |
|  | Lymphocyte (%) | 82.45 ± 0.70 | 83.30 ± 1.35 | 83.43 ± 1.96 |

**Table S2** The effect of AADF on haematological parameters (neutrophil and lymphocyte). The mice were treated with saline or AADF for seven days, respectively. The haematological parameters were tested at 3th and 7th day.


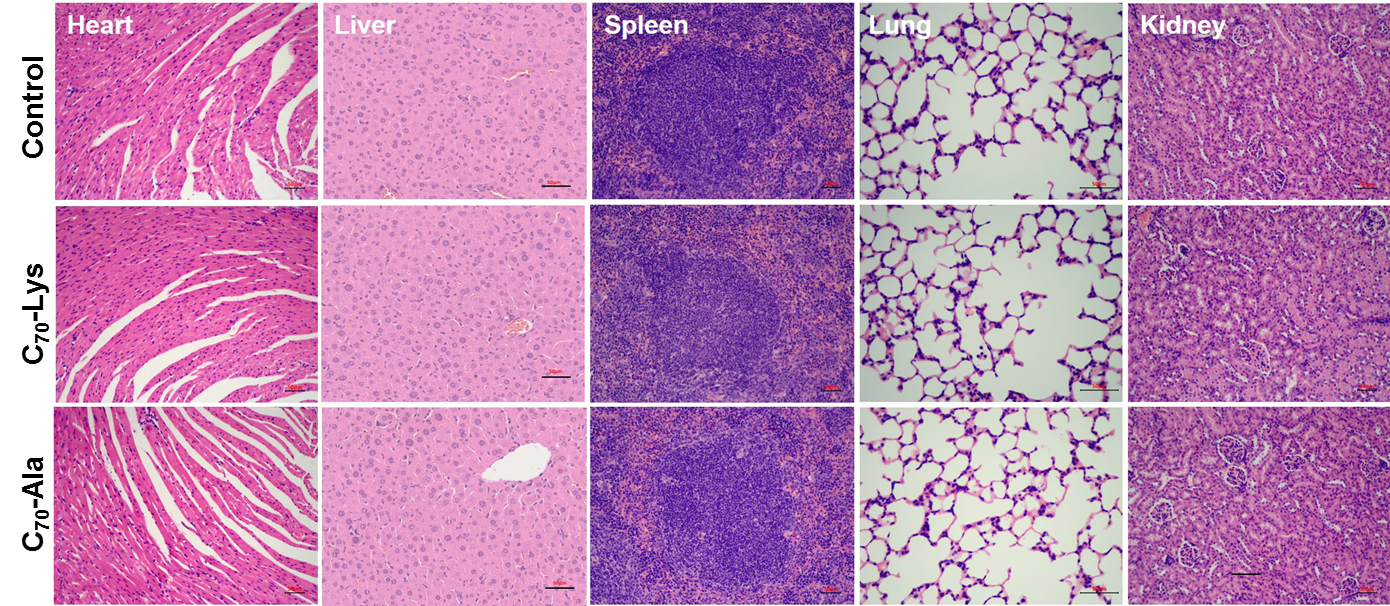


**Figure S7** The histologic sections of the main organs in the control, C_70_-Lys and C_70_-Ala groups.

| Day | Parameter | Group | | | |
| --- | --- | --- | --- | --- | --- |
|  |  | Control | DOX+C_70_-Ala (L) | DOX+C_70_-Ala (M) | DOX+C_70_-Ala (H) |
| Pre | Neutrophil (%) | 18.45 ± 2.05 | 19.22 ± 2.10 | 20.00 ± 3.50 | 20.37 ± 2.44 |
|  | Lymphocyte (%) | 79.65 ± 2.35 | 79.80 ± 2.72 | 78.10 ± 2.72 | 78.40 ± 3.23 |
| Post-2 h | Neutrophil (%) | 16.37 ± 2.40 | 43.80 ± 3.32 | 31.30 ± 3.58 | 30.53 ± 1.86 |
|  | Lymphocyte (%) | 81.17 ± 3.95 | 54.67 ± 2.22 | 67.07 ± 3.09 | 67.80 ± 1.22 |

**Table S3** The short-term effect of AADF on neutrophil and lymphocyte. The mice were pre-treated with saline or AADF at different concentrations for three days, respectively. DOX was *i.v.* injected on the 4th day and the haematological parameters were tested after two hours.

| Coefficient  (mg/g) | Group | | | |
| --- | --- | --- | --- | --- |
|  | Control | DOX+C_70_-Ala (L) | DOX+C_70_-Ala (M) | DOX+C_70_-Ala (H) |
| Liver | 44.69 ± 1.34 | 53.36 ± 2.29 | 51.50 ± 4.14 | 50.13 ± 1.19 |
| Heart | 4.93 ± 0.28 | 5.32 ± 0.50 | 5.26 ± 0.41 | 5.21 ± 0.35 |

**Table S4** The coefficients of liver and heart after sacrificing.


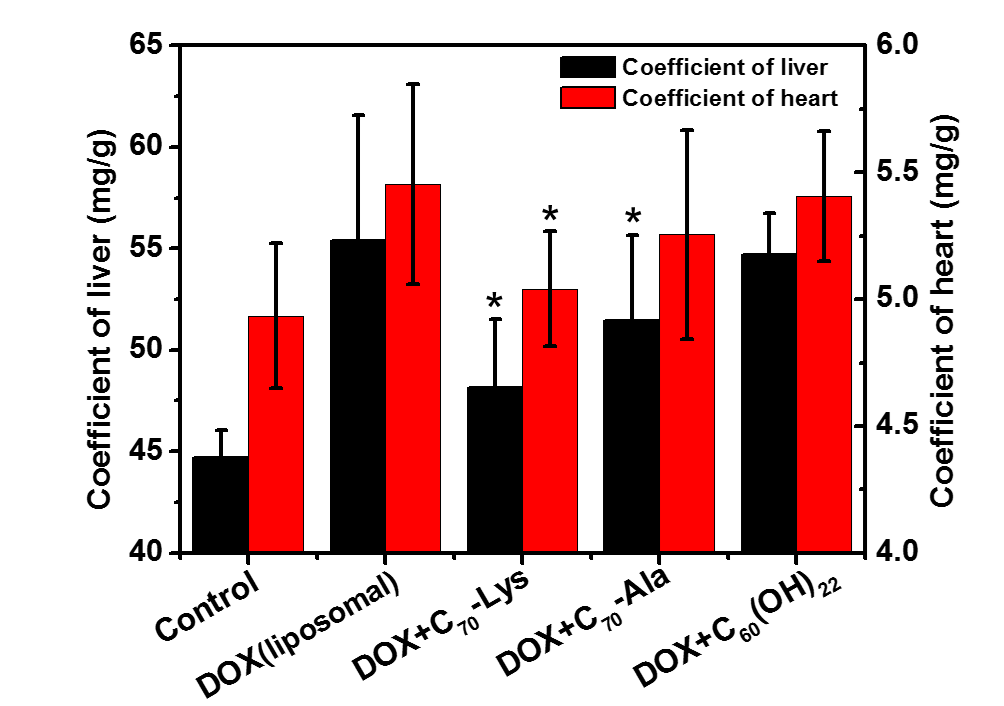


**Figure S8** The coefficients of liver and heart after sacrificing. ^*^*P*<0.05 *vs.* DOX (liposomal) group.


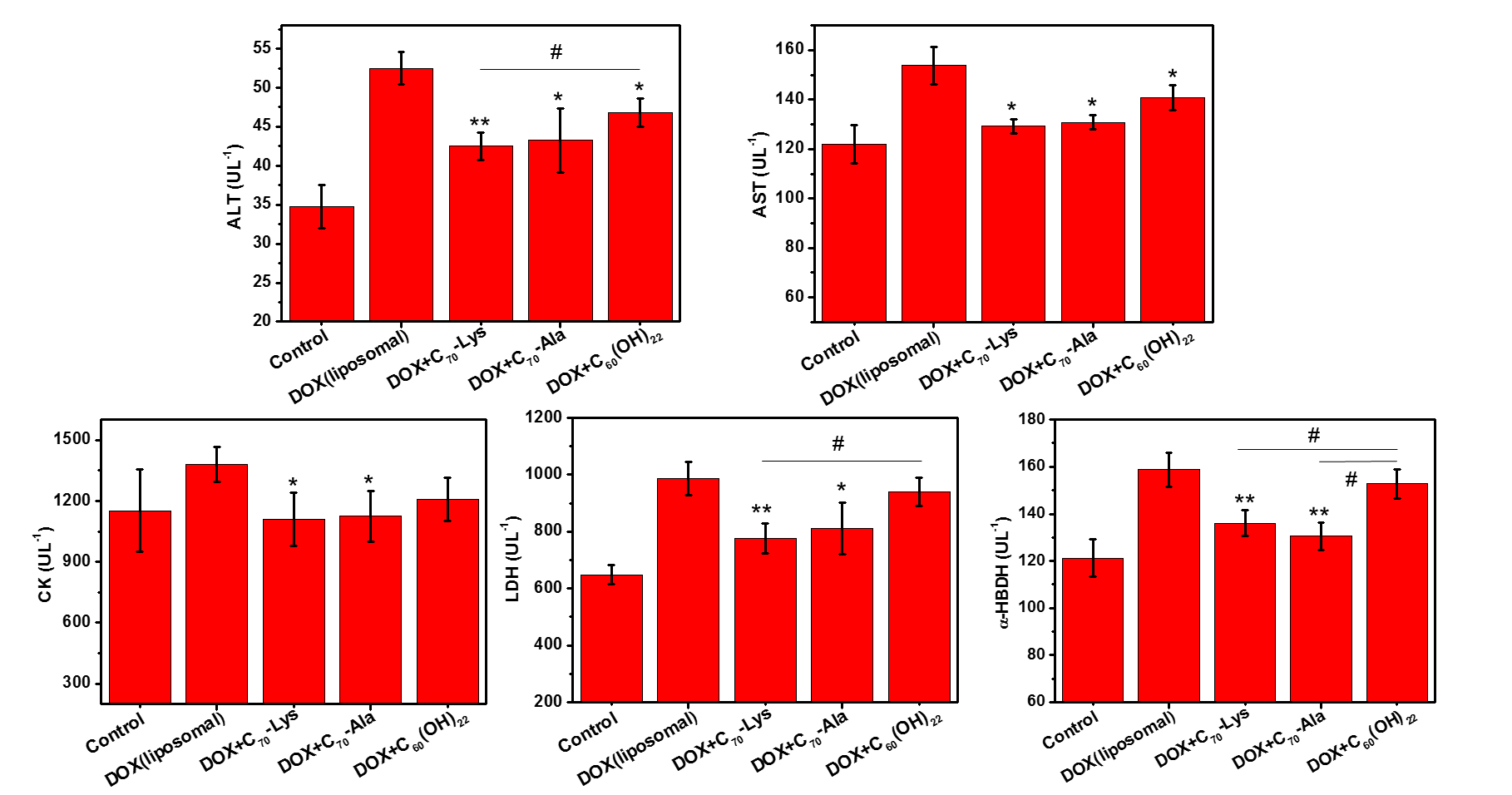


**Figure S9** Serum ALT, AST, CK, LDH, α-HBDH levels of different groups. ^*^*P*<0.05 and ^**^*P*<0.01 *vs.* DOX (liposomal) group; ^#^*P*<0.05 *vs.* DOX + C_60_(OH)_22_ group.

**Biomarker assay of liver and heart tissues:** The liver and heart were minced and homogenized in a Tris-buffer solution (pH 7.4; organ: buffer 1:10; w/w) and divided into two portions. One was used for MDA determination, and the other was centrifuged to obtain the supernatant for the assays of total protein content, LDH, GSH, GSSG, GSH-Px, GR, CAT and SOD.


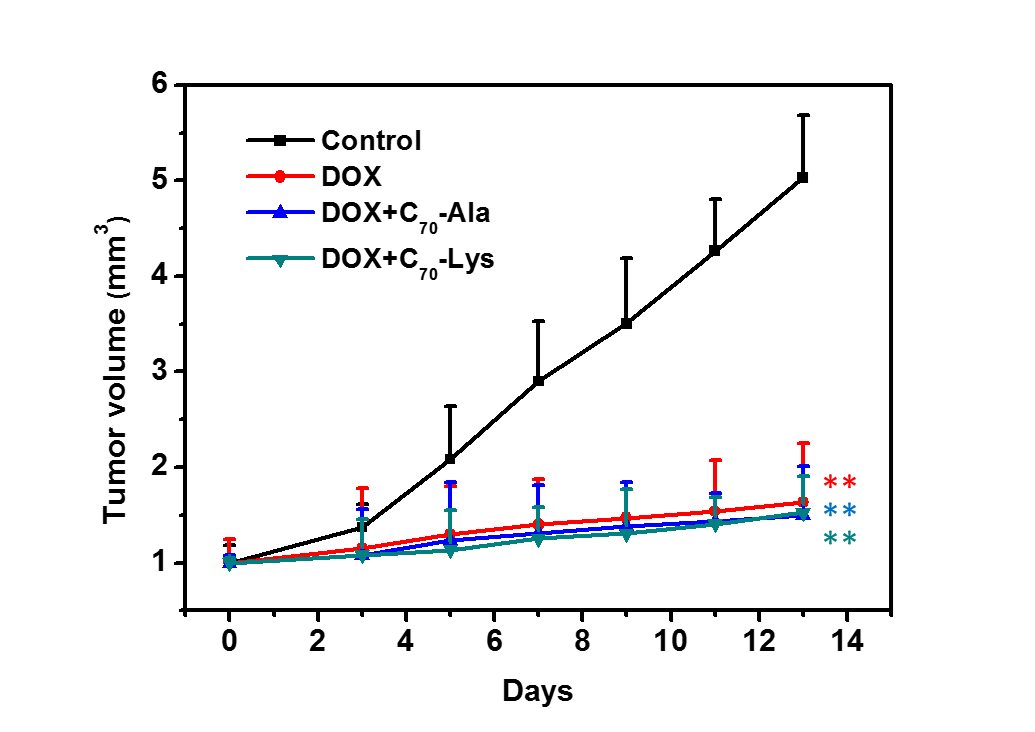


**Figure S10** The therapy effect of different groups on H22 tumor. The growth inhibitory curves of tumors in the control group, DOX group, DOX + C_70_-Ala group and DOX + C_70_-Lys group (n = 6; ^**^*P*< 0.01, compared with the control group). DOX was *i.v.* injected at a dose of 10 mg/kg every three days and C_70_-Ala/ C_70_-Lys were *i.p.* injected at a dose of 10 mg/kg daily.

**Western blotting:** In brief, protein samples were subjected to SDS-polyacrylamide gel electrophoresis (SDS-PAGE) and transferred onto a polyvinylidene difluoride membrane (Bio-Rad, USA). After blocking for 60 min with 2% BSA, membranes were incubated with the primary antibody (anti-CYP2E1 antibodies: Abcam, Inc., Cambridge, MA, USA) overnight at 4°C followed by incubation with the corresponding secondary antibody (GE healthcare) for 60 min at room temperature. Membranes were visualized with an ECL-plus detection system (GE Healthcare, NJ).


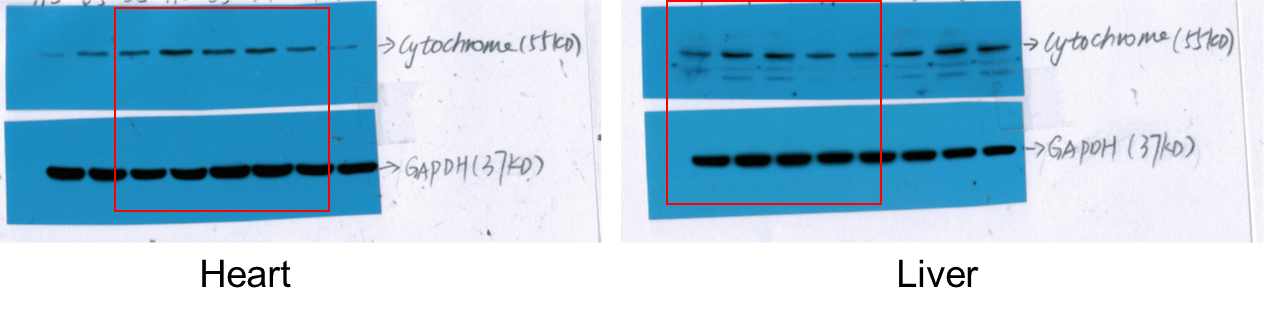


**Figure S11.** Original gel pictures associated with Figure 6 indicated.
